# Involuntary Weight Loss and Late-Life Depression in Korean Older Adults

**Published:** 2020-04

**Authors:** Youngyun JIN, Jinkyung CHO, Inhwan LEE, Soohyun PARK, Donghyun KIM, Jiyoung KONG, Hyunsik KANG

**Affiliations:** College of Sport Science, Sungkyunkwan University, Suwon, Republic of Korea

**Keywords:** Involuntary weight loss, Mental health, Older adult, Covariates

## Abstract

**Background:**

To investigate the association between involuntary weight loss (IWL) and late-life depression (LLD) in a population-based cohort study.

**Methods:**

Data (N=6945) obtained from the 2008 baseline and 2011 follow-up assessments of the Living Profiles of Older People Survey in Korea were used. Changed body weight between the 2008 and 2011 was classified into stable weight (<−5% ∼ <+5%), lightweight loss (≥ −5% ∼ <−10%), moderate weight loss (≥ −10%), lightweight gain (+≥5 ∼ <+10%), and moderate weight gain (≥+10%).

**Results:**

Compared to the stable weight group, the moderate weight loss group had a higher risk of LLD (odds ratio=1.99, 95% confidence interval=1.61–2.46, *P*<0.001) even after adjustments for covariates, including age, BMI, education, income, life of solitude, smoking, physical activity, dependent aging, comorbidity, and cognitive function.

**Conclusion:**

IWL is significantly associated with LLD in Korean older adults, implying the prognostic importance of IWL for presenting mental health issues later in life.

## Introduction

Involuntary weight loss (IWL) is defined as a 5% reduction in body weight within 6–12 months (1) and is a common experience among older adults, with an annual incidence of approximately 15% to 20% (2). The clinical consequences of IWL include functional decline (3), decline in activities of daily living (4), infections (5), increased use of acute and long-term care facilities (6), and the presenting mental disorders such as late-life depression (LLD) (7).

LLD is one of the most prevalent psychiatric disorders in older adults and is associated with greater cognitive and functional decline and dementia as well as increased morbidity and mortality (8,9). Despite its prevalence and clinical significance, however, LLD is under-recognized and under-treated due to its complicated etiologies, and being mistaken as problems of aging, implying the imperativeness of an effective and proper treatment for LLD and its clinical consequences.

With a rapidly aging society, geriatric mental health is emerging as an important public health concern in Korea and has a serious impact on quality of life. For example, depression is one of the most significant public health problems in Korea. Prevalence of major depressive disorders in Korean older adults is estimated to range from 3.7% to 11.0% (10), which is higher than those of Western countries (11). The majority of sufferers (14.9∼37.0%) were over 60 yr with higher prevalence in women than in men (10). Furthermore, the prevalence of depressive symptoms in Korean older adults increases with aging, reaching its peak of 35.4% at age of 80 yr and older (12). Yet, limited information is available regarding IWL and its relation to LLD in Korean older adults. The etiology of LLD in Korea is still inconclusive (13), partly due to limited number of previous studies (12). In this population-based cohort study, therefore, we aimed to investigate the association between IWL and LLD in Korean older adults aged 60 yr and older.

## Methods

### Study sample (data source)

Data from the 2008 and 2011 Living Profiles of Older People Survey (LPOPS) were used for this study. The LPOPS was conducted every 3 years among people aged 60 yr and older living in Korea to collect basic data and indices required to establish policies for older people.

The baseline assessment, first survey, was conducted in 2008, and a follow-up assessment, second survey, was conducted in 2011. Initially, 19007 older people aged 60 yr and older were invited to the 2008 LPOPS, and 15146 completed the baseline assessments (the response rate of 79.7%). After the baseline assessments, 2884 subjects were excluded due to diagnosed depression. Additional 5308 subjects did not complete the assessments due to deaths (N=902), refusal or loss of contact (N=4241), and missing data in body weight (N=165) during the 2011 follow-up survey, leaving the final sample size of 6954 subjects for this study.

The Sungkyunkwan University Institutional Review Board, in accordance with the World Medical Association Declaration of Helsinki, reviewed and approved the study protocol (SKKU 2017-06-009).

### Determination of Depression (Exposure)

Depression was the exposure of interest in this study. The Korean version of the short form of the Geriatric Depression Scale (SGDS-K), a shortened form of the Geriatric Depression Scale (14), was administered as a screening measure for depression in this study population. This is a 15-item self-report binary response format (yes/no) with a range of scores from 0 to 15. Severity was assessed by SGDS scores. Depression was defined when diagnosed by a physician with a score of >8 on the SGDS-K. The SGSD-K was previously validated in registered elderly Korean psychiatric patients aged 55 yr and older (15).

### IWL (Exposure)

Weight was measured at baseline and at follow-up. IWL was defined as 5% or more weight loss in the last 30 d without an obvious cause (or 10% or more in the last 180 d) as compared to baseline weight (16). Then, weight change occurred during 3-year follow-up period (difference between at baseline and at follow-up) was calculated and classified into 3 weight change groups: loss (≥5% of loss), stable (within ±5% loss or gain), and weight gain (≥5% of gain). Subjects were excluded for voluntary weight loss (i.e., a diet), accelerated metabolism, increased caloric loss in urine or stool, known malignancy, or availability of clinical diagnostic information that enabled the diagnosis of the cause of weight loss (6).

### Covariates

Height was assessed using a measuring tape with the participant standing with the back of the head, scapulae, buttocks, and heels in contact with a vertical board. Body weight was measured to the nearest 0.1 kg with a portable digital scale, after removing the shoes and wearing only light clothing. BMI was calculated by dividing body weight by height squared (kg/m^2^).

Covariates assessed in the study included socio-demographic factors including age, gender, education (years), monthly income, living status (i.e., alone or with family), and marital status (i.e., never married or married or widowed or divorced/separated). Additionally, health behavioral factors were measured including current smoking and alcohol consumption, nutritional status, number of comorbidity, disability, cognitive impairment, and physical activity. Smoking status was categorized as non-smoker, past smoker, or current smoker. Alcohol consumption was classified as abstinent, no drinker, ≤1 time per week, and ≥2 times per week. Nutritional score was assessed using the nutrition screening initiative checklist (17). Comorbidity was defined as the number of physician-diagnosed chronic conditions (hypertension, stroke, angina, diabetes mellitus, arthritis, chronic bronchitis/emphysema, asthma, cancer, chronic renal failure, and fracture). Disability was measured using the Korean version of the Instrumental Activities of Daily Living Scale (K-IADL) (18). Cognitive function was assessed using the Korean version of the Mini-Mental State Examination (MMSE-KC) (19).

### Statistical analyses

Means and standard deviations were computed for continuous variables, while proportions and percentages were calculated for categorical variables. Independent t-test and chi-square tests were used to compare baseline values of continuous and categorical variables, respectively. To assess the risk of IWL for incidence of LLD, changed body weight between the 2008 baseline and 2011 follow-up measurements was calculated and classified into 5 categories based on a clinically relevant amount of body weight (16); stable weight (<−5% ∼ <+5%), lightweight loss (≥ −5% ∼ <−10%), moderate weight loss (≥ −10%), lightweight gain (+≥5 ∼ <+10%), and moderate weight gain (≥+10%). The Kruskal-Wallis test was used to test any significant linear trends in the incidence rates of LLD according to levels of weight change. Odds ratios (ORs) and 95% confidence intervals (95% CIs) for LLD were estimated according to the weight change-based categories using multiple logistic regression analyses before and after adjustments for the covariates. Alpha was set at 0.05. SPSS statistical software, ver. 21.0 (Chicago, IL, USA) was used to perform all statistical analysis.

## Results

Table 1 represents the description of study participants with respect to measured variables. Women were heavier than men (*P*<0.001), with no gender difference in mean age (*P*=0.543). Men had higher levels of education (<0.001) and monthly income (<0.001) than women. Men had higher rates of smoking (<0.001) and alcohol consumption (<0.001) than women. Men had higher levels of physical activity (*P*<0.001) and no gender difference in social activity (*P*=0.508). Women had higher rates of life of solitude (*P*<0.001), lower rates of poor nutritional status (*P*<0.001), higher K-IADL (*P*<0.001) scores, number of comorbidities (*P*<0.001) and lower MMSE-KC scores (*P*<0.001) than men.

**Table 1:** Descriptive statistics of study participants

***Variable***	***Total (N=6,954)***	***Men (N=3,070)***	***Women (N=3,884)***	**P *value***
Age (yr)	73.3±6.2	73.2±6.0	73.3±6.4	0.543
BMI (kg/m^2^)	23.7±3.1	23.3±2.9	24.1±3.3	<0.001
Education (years)	6.2±4.7	8.4±4.4	4.4±4.0	<0.001
Monthly income (1000 won)	1536±1772	1680±1862	1422±1689	<0.001
Past/Current smoking	2,328(33.5)	2,143(69.8)	185(4.8)	<0.001
Alcohol consumption				
No drinking	4,356(62.7)	1,284(41.8)	3,072(79.1)	
≤1 times per week	1,343(19.3)	706(23.0)	637(16.4)	
≥2 times per week	1,252(18.0)	1,079(35.2)	173(4.5)	<0.001
Social activity				
No participation	1,100(15.8)	496(16.2)	604(15.6)	
Participation	5,854(84.2)	2,574(83.8)	3,280(84.4)	0.508
Life of solitude				
With family	3,789(75.7)	2,133(92.3)	1,656(61.4)	
Alone	1,219(24.3)	178(7.7)	1,041(38.6)	<0.001
Physical activity				
No participation	5,714(82.2)	2,352(76.6)	3,362(86.6)	
Participation	1,240(17.8)	718(23.4)	522(13.4)	<0.001
Nutritional status (score)	2.5±2.6	2.2±2.4	2.7±2.7	<0.001
K-IADL (score)	10.4±1.6	10.3±1.5	10.5±1.7	<0.001
Comorbidity (number)	1.8±1.5	1.5±1.3	2.1±1.5	<0.001
MMSE-KC (score)	24.6±3.7	25.9±3.1	23.6±3.8	<0.001

BMI: body mass index; K-IADL: the Korean version of the Instrumental Activities of Daily Living Scale MMSE-KC: Korean version of the Mini-Mental State Examination

Table 2 represents the incidence rates of LLD during the 3-year follow-up period in this study population. Incidence rate of LLD was 17.9% in the total study population, 20.0% in women, and 15.7% in men, with its significantly higher incidence rate (*P*<0.001) in women than in men. In addition, there were significant differences in the incidence rates of LLD according to levels of weight change.

**Table 2:** Incidence rates of 3-year-late-life depression according to levels of weight change

***Variable***	***SW (N=3,969)***	***LWG (N=441)***	***LWL (N=1,374)***	***MWG (N=352)***	***MWL (N=818)***	**P *for linear trends***
Total, N (%)	710 (17.9)	106 (24.0)	312 (22.7)	96 (27.3)	280 (34.2)	<0.001
Men, N (%)	299 (15.7)	40 (19.2)	93 (17.6)	28 (20.0)	99 (34.7)	<0.001
Women, N (%)	411 (20.0)	66 (28.3)	219 (25.9)	68 (32.1)	181 (34.0)	<0.001

SW: stable weight; LWG: light weight gain; LWL: light weight loss; MWG: moderate weight gain; MWL: moderate weight loss

There were significant positive, linear trends in the incidence rates of 3-year-cumulative depression in the total (*P*<0.001), men (*P*<0.001), and women (*P*<0.001) according to levels of weight change (from stable weight to lightweight loss, lightweight gain, moderate weight gain, and moderate weight loss in order). In the total study population, the incidence rate of LLD was highest in the moderate weight loss group (34.2%), followed by moderate weight gain (27.3%), lightweight gain (24.0%), and lightweight loss (22.7%), and stable weight group (17.9%) in order. In men, the incidence rate of LLD was highest in the moderate weight loss group (34.7%), followed by the moderate weight gain (20.0%), moderate weight gain (19.5%), lightweight loss (17.6%), and stable weight group (15.7%) in order. In women, the incidence rate of LLD was also highest in the moderate weight loss group (34.0%), followed by the moderate weight gain (32.1%), lightweight gain (28.3%), lightweight loss (25.9%), and stable weight group (20.0%) in order.

Table 3 represents the odds ratios (ORs) of LLD according to levels of weight change. The moderate weight loss (OR=2.14, *P*<0.001), moderate weight gain (OR=1.58, *P*<0.001), lightweight loss (OR=1.28, *P*=0.002), and lightweight gain groups (OR=1.42, *P*=0.004) were at significantly higher risks of LLD after adjustment for age, as compared to the stable weight group (referent, OR=1). The moderate weight loss group (OR=2.02, *P*<0.001) or the moderate weight gain group (OR=1.49, *P*=0.011) had a significantly increased risk of LLD after additional adjustments for education, income, and life of solitude. Furthermore, the moderate weight loss group had an increased risk of LLD (OR=1.99, *P*<0.001) even after additional adjustments for BMI, physical activity, smoking, number of comorbidities, K-IADL, and MMSE score.

**Table 3:** Odds ratios and 95% confidence intervals for 3-year-late-life depression according to levels of weight change

***Variable***	***SW***	***LWG***	**P**	***LWL***	**P**	***MWG***	**P**	***MWL***	**P**
Model 1	Referent	1.42 (1.12–1.79)	0.004	1.28 (1.10–1.49)	0.002	1.58 (1.22–2.03)	<0.001	2.14 (1.81–2.54)	<0.001
Model 2	Referent	1.25 (0.93–1.68)	0.148	1.17 (0.97–1.40)	0.098	1.49 (1.10–2.04)	0.011	2.02 (1.66–2.48)	<0.001
Model 3	Referent	1.22 (0.90–1.66)	0.208	1.16 (0.96–1.41)	0.125	1.34 (0.96–1.87)	0.081	1.99 (1.61–2.46)	<0.001

SW: stable weight; LWG: light weight gain; LWL: light weight loss; MWG: moderate weight gain; MWL: moderate weight loss.

Model 1 adjusted for age and sex.

Model 2 adjusted for Model 1 plus education, monthly income, social activity, and life of solitude.

Model 3 adjusted for Model 2 plus body mass index, physical activity, smoking, nutritional status, disability, comorbidity, and cognitive function.

## Discussion

In this population-based cohort study, we examined the association between IWL and LLD during the 3-year-follow-up period in Korean elderly persons aged 60 yr and older. We found that ≥ 10% IWL was significantly associated with an increased risk of LLD in the Korean geriatric population, and the increased risk of ≥10% IWL for LLD remained significant even after adjustments for the covariates assessed in the study, including age, BMI, physical activity, smoking, number of comorbidities, K-IADL, and MMSE score. Together, the findings suggested that IWL is an independent predictor of LLD in Korean older adults. The current findings are in agreement with previous studies reporting a significant association between IWL and depression and/or depressive symptoms. In a prospective study of heart failure older patients in Korea, cardiac patients who experienced ≥6% IWL had an increased risk of depressive symptoms in conjunction with 3.2 times higher risk of cardiac events (20). In the Health, Aging and Body Composition (Health ABC) Study, depressed mood predicted weight gain, while weight loss predicted depressed mood in older adults (21). Depression was independently associated with weight loss in community-dwelling older adults (22). In another prospective study of a nursing home population, psychiatric and psychological diseases were one of the primary reasons for unexplained weight (23). Together, those findings including the current one suggest that IWL is significantly associated with depression and/depressive symptoms in older adults. Yet, the possibility of the bidirectional relationship between the two health issues cannot be ruled out.

Several explanations can be given to mechanism for IWL associated with LLD. First, inadequate food intake (3), a worsening of chronic diseases (3), loss of appetite and consequent anorexia due medication use (2) and polypharmacy (24), non-malignant gastrointestinal disease (25), and others are known as physiological factors for IWL. Second, psychiatric disorders such as depression and dementia (22) may be another reason for IWL in older adults. Lastly, social factors, including poverty, alcoholism, isolation, financial constraints, and other barriers to obtaining food might be additional reasons for IWL in older adults (2). Together, it seems most plausible that all the above mentioned physiological, psychological, and social factors are collectively responsible for the mental health issue in older adults. However, it is unclear whether IWL is a direct cause or a marker for those underlying conditions in the current study.

The present study had several strengths. Study participants of Korean older adults were recruited by stratified two-stage cluster sampling, and the overall response rates of 79.7% and 66.0% at baseline and at 3-year follow-up, respectively, were relatively high. Many covariates as possible were assessed in order to obtain a more reliable and reproducible association between the exposure and outcome. However, the study also had some limitations. First, the cross-sectional nature of this population-based prospective study does not allow for any causal inference regarding the link between IWL and depression. Thus, the current findings remain to be confirmed in a cause-effect manner. Second, biomarker data, such as inflammatory cytokines, which might influence the association between weight change and LLD, were not available in this study. Although age was controlled as a covariate in our analysis due to limited sample size of old-old adults aged 85 yr and older (i.e., approximately 4%), it may be possible that weight change may differently influence incidence of LLD between old (aged 65 ∼84 yr) and old-old (aged 85 yr and older) populations. Therefore, we cannot be certain that our findings would apply equally to old-old population in Korea. Third, caution is needed regarding the generalization of the current findings because the number of individuals excluded is relatively large. Lastly, IWL may be a clinical outcome of chronic conditions in older adults (1), and it remains to be further explored.

IWL is a common condition among older adults in Korea. In addition to LLD, the clinical consequences of IWL if untreated properly would be detrimental, including functional decline, the declines in physical fitness and function (26,27), increased risks of metabolic diseases (28,29), infections (6), decubitus ulcers (1), exacerbation of cognitive and mood disorders (22,23), increased use of acute and long-term care facilities (24), and increased morbidity and mortality (30).

## Conclusion

IWL is significantly associated with LLD in Korean older adults, implying the prognostic importance of IWL for presenting mental health issues later in life.

## Ethical considerations

Ethical issues (Including plagiarism, informed consent, misconduct, data fabrication and/or falsification, double publication and/or submission, redundancy, etc.) have been completely observed by the authors.
